# Exploring human papillomavirus vaccine hesitancy among college students and the potential of virtual reality technology to increase vaccine acceptance: a mixed-methods study

**DOI:** 10.3389/fpubh.2024.1331379

**Published:** 2024-02-13

**Authors:** Sangchul Yoon, Heeyeon Kim, Juhyeong An, Seok Won Jin

**Affiliations:** ^1^Department of Medical Humanities and Social Sciences, College of Medicine, Yonsei University, Seoul, Republic of Korea; ^2^Center for Global Development, Yonsei lnstituite for Global Health, Yonsei University Health System, Seoul, Republic of Korea; ^3^School of Social Work, The University of Memphis, Memphis, TN, United States; ^4^Institute of Media Arts, Yonsei University, Seoul, Republic of Korea

**Keywords:** college students, HPV vaccination, human papillomavirus, mixed-methods study, vaccine hesitancy, virtual reality

## Abstract

**Background:**

Human papillomavirus (HPV) can cause cancers in men and women. Despite the availability of an effective vaccine, HPV vaccination coverage remains suboptimal among college students. Literature showed that hesitancy for HPV vaccination is a leading barrier to the uptake in this group. However, prior interventions have shown limitations in reducing HPV vaccine hesitancy in college students. Thus, this study examined a conventional educational approach using a vaccine information statement (VIS), and subsequently explored college students’ HPV vaccine hesitancy and the potential of virtual reality (VR) technology to overcoming the limitations of interventional efforts.

**Methods:**

We employed a mixed-methods design along with convenience sampling, constituting a one-way pre- and post-intervention (HPV VIS) survey (Study A) and individual interviews (Study B). All data collections occurred with 44 college students at an urban public university at the mid-south region of the U.S. between October 2022 and April 2023. Study A assessed changes in HPV vaccination outcomes including knowledge, beliefs/attitudes, vaccine hesitancy, and intentions. Study B measured college students’ primary reasons for HPV vaccine hesitancy and preferred strategies for the vaccination promotion including VR-based education. We conducted paired t-test and Wilcoxon signed ranks test for quantitative data and framework analysis for qualitative data.

**Results:**

Participants reported significant improvements in knowledge [*t*(43) = 6.68, *p* < 0.001] regarding HPV vaccination between before and after reading the HPV VIS. No change was observed in beliefs/attitudes, vaccine hesitancy, and intentions. The framework analysis revealed college students’ reasons for HPV vaccine hesitancy, needed information, and preferred strategies along with the potential of VR technology for future HPV vaccination education.

**Conclusion:**

The findings provided essential information on designing HPV vaccination information focused on vaccine hesitancy among college students. Future research should consider these findings in developing interventions including VR to increasing HPV vaccine acceptance among college students.

## 1 Introduction

Human papillomavirus (HPV) is the most common sexually transmitted infection in the United States (U.S.) (1). Among over 200 varieties of this virus, long-lasting infections with high-risk HPVs (e.g., HPV16 and HPV18) can lead to cancers for both women and men (2). HPV accounts for the majority of cervical cancers, above 90% of anal cancers, 75% of vaginal cancers, 70% of oropharyngeal and vulvar cancers, and over 60% of penile cancers in the U.S. (2). It is estimated that there were approximately 47,199 new cases of HPV-associated cancer in the U.S. each year, with 26,177 cases in women and 21,022 cases in men (3).

The HPV vaccine is highly effective in protecting against the types of HPV which lead to cancer (4). The Advisory Committee on Immunization Practices (ACIP) recommends initiating HPV vaccination as early as age 9, and receiving routine vaccination at age 11 or 12, or catch-up vaccination for adults up to the age of 26 who have not yet received their recommended vaccinations (5). Although safe, efficacious vaccines against HPV have been available since 2006 for women and 2009 for men (6, 7), many eligible young adults have chosen to delay or refuse HPV vaccination (8–10). Across all age-based populations in the U.S., college students have reported high rates of HPV prevalence along with low rates of HPV vaccination (11–13). The 2022 National College Health Assessment survey demonstrated that only 41% of male college students and 57% of female college students were up to date with recommended HPV vaccine series (14). This lags behind 60 and 64% of HPV vaccination coverage for male and female adolescents, respectively, aged 13 through 17 years (15), and falls far short below the 80% benchmark of the national goal set by Healthy People 2030 (8–10).

Existing literature has shown that the suboptimal HPV vaccination coverage in U.S. college students was associated with multi-level factors: individual characteristics, interpersonal influences, and social and structural factors. Individual characteristics include a lack of awareness and knowledge of HPV and the vaccination (16) and HPV vaccine-related misperceptions, misbeliefs, and negative attitudes (17, 18). Interpersonal influences include parent’s or peer’s negative attitudes toward the vaccination or vaccine endorsement (19, 20), and lack of recommendations from healthcare providers (17). Social and structural factors include social/religious norms or stigma (11, 19, 21, 22), the spread of misinformation (19, 23), costs and insufficient insurance coverage (20, 21, 24), complex vaccination schedules (24), limited campus vaccine availability, and the absence of policies or programs for HPV vaccination (22).

Research also has indicated that vaccine hesitancy is a leading barrier to HPV vaccination among college students (25, 26). Further, over the past few years the COVID-19 pandemic has elevated levels of vaccine hesitancy both nationally and globally (27). The Strategic Advisory Group of Experts on Immunization Working Group (hereafter, Sage Working Group), established by the World Health Organization (WHO), defined vaccine hesitancy as a “delay in acceptance or refusal of vaccination despite availability of vaccine services” (28). Vaccine hesitancy is characterized by its complexity, context specificity, and variabilities based on time, place, and types of vaccines, existing on a continuum between full acceptance and outright refusal of vaccination (29).

To increase HPV vaccination among college students, previous interventions have employed health education and communication strategies. These strategies involve one or a combination of: (a) delivery of HPV vaccination information or messages by peers and/or health experts (30, 31) via leaflets (32), videos (33), websites (34, 35), text/email (36), social media (37), or campaigns (38, 39) and (b) reminders of vaccine appointments using letters (40) or electronic messaging (36, 41). These interventional efforts yielded some positive vaccination outcomes, with improvements mostly observed in knowledge of HPV and the vaccination (23, 36), some in HPV vaccination-related perceptions, beliefs, attitudes, and/or intentions (23, 24, 42), but only limited increases in the uptake (20, 42). To date, few interventions have demonstrated rigorous evidence on their effectiveness in increasing actual uptake of HPV vaccination among college students. According to literature, one reason for this is because the majority of the interventions focused predominantly on increasing knowledge of HPV and the vaccination, but increased knowledge alone was insufficient to close the knowledge-vaccine adoption gap (24). Another reason is that the interventions addressed college students’ hesitancy for HPV vaccination only for certain dimensions of the hesitancy (43–45), while the vaccine hesitancy constitutes complex, multi-dimensional constructs (26). According to the SAGE Working Group (29), vaccine hesitancy comprises three psychological dimensions: confidence (trust in the effectiveness of a vaccine), complacency (perceptions of the need to vaccinate and risk), and convenience (accessibility, self-efficacy), categorized at multiple levels (including contextual influences, individual/group influences, and vaccine/vaccination-specific issues). Also, a recent systematic review conceptualized vaccine hesitancy as a state of indecisiveness regarding a vaccination decision, determined by two psychological factors: cognitive factors (knowledge, beliefs, self-efficacy) and affective factors (emotion, attitudes) (46). Therefore, there is a need for developing an innovative, effective intervention that overcomes the limitations of prior efforts to close the knowledge–behavior gap in HPV vaccine practice by reducing the vaccine hesitancy among college students through addressing multi-dimensions of the vaccine hesitancy.

An increasing body of research has attended to virtual reality (VR) to facilitate vaccination, widely employing it in the sectors ranging from health communication to medical training (47). VR is an interactive three-dimensional simulation which permits a person to experience real-world environments and learn new behaviors and skills through avoiding obstacles to targeted outcomes (48). Emerging evidence has demonstrated the potential of VR as an effective health education tool for addressing vaccine hesitancy and positively influencing a person’s vaccine-related decision-making (43). However, no study has examined the role of VR in lowering hesitancy for HPV vaccination especially for college students to advance their vaccination outcomes.

To develop a VR-based approach to alleviating hesitancy for HPV vaccination among college students and increasing their vaccination coverage, it is essential to understand why college students are hesitant to receive an HPV vaccine and what information they need to address the hesitancy. It is also important to obtain data on how college students view VR as a health education platform to deliver the vaccination information. Moreover, although HPV vaccine information statements (VIS) have been widely used in health education campaigns for the public. The functioning of the HPV VISs concerning HPV vaccination outcomes, including knowledge, beliefs, attitudes, intentions, and vaccine hesitancy among college students, remains unknown. Thus, this study employed a mixed methods design consisting of quantitative pre- and post-intervention survey (Study A) and qualitative interviews (Study B). Study A aimed to evaluate whether a conventional HPV vaccination education using an existing HPV vaccine information statement (HPV VIS, provided by the Centers for Disease Control and Prevention [CDC]) (49) can improve HPV vaccination outcomes including knowledge, beliefs/attitudes, vaccine hesitancy, and intentions among college students. Study A hypothesized that after reading the HPV VIS, college students would report significant changes in knowledge, beliefs/attitudes, vaccine hesitancy, and intentions compared to their status before reading the HPV VIS. The findings would inform the design and development of educational content aimed at reducing HPV vaccine hesitancy and facilitating the uptake among college students.

Additionally, Study B aimed to explore college students’ reasons for HPV vaccine hesitancy, needed information directed toward HPV vaccine hesitancy and the vaccination promotion, and preferred strategies and the potential of VR for improving HPV vaccination acceptance. The research questions of Study B include:

Research question 1: What are primary reasons for HPV vaccine hesitancy among college students?

Research question 2: What specific information do college students need to reduce HPV vaccine hesitancy and make an informed decision about HPV vaccination?

Research question 3: What are college students’ preferred strategies for HPV vaccination promotion and views of the potential of VR for the vaccination education?

## 2 Materials and methods

### 2.1 Study design and procedure

**Figure 1:**
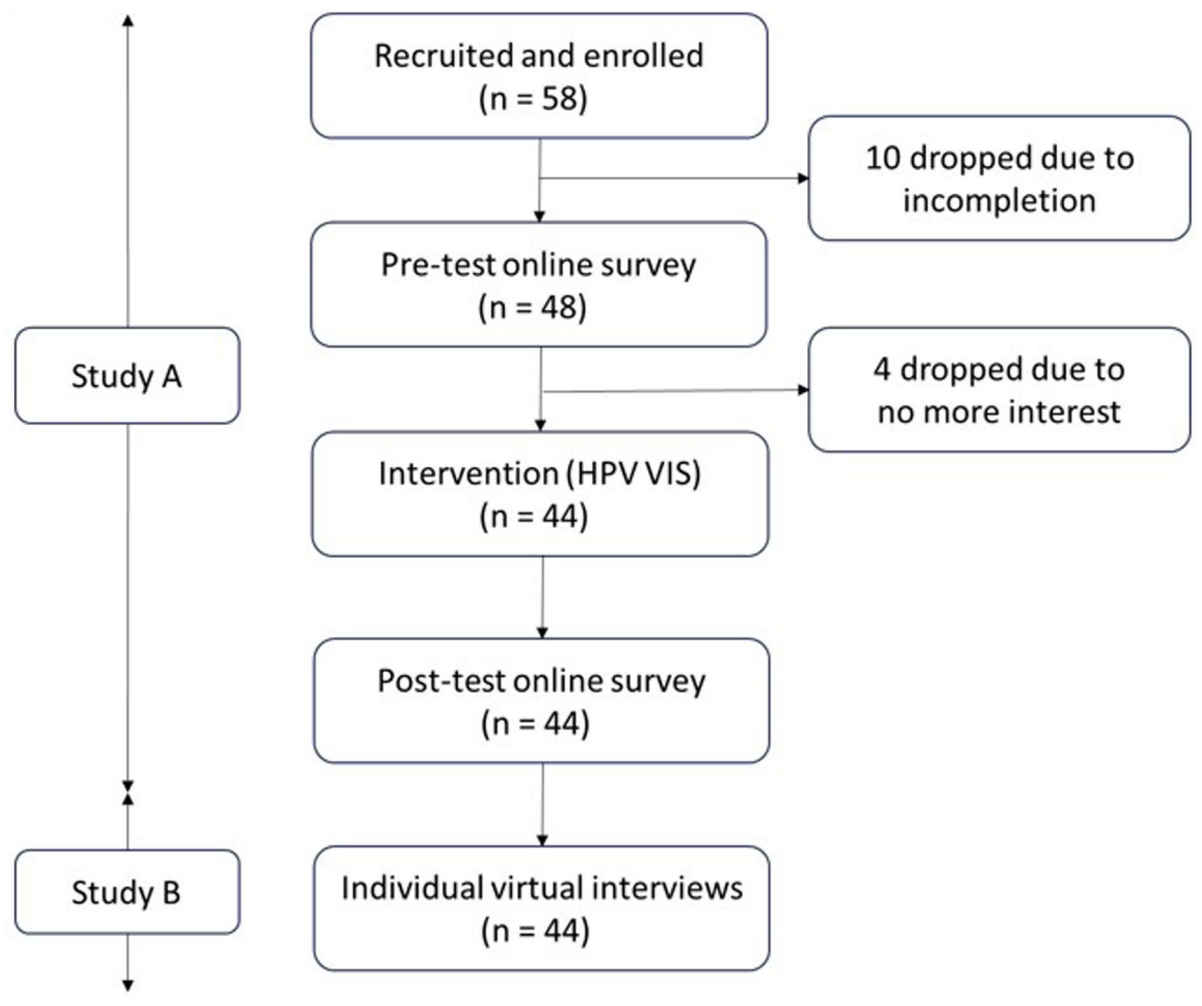
Study design and procedure.

As shown in Figure 1, we employed a sequential quantitative-qualitative mixed method study with an emphasis on qualitative research—i.e., a quan-QUAL design (50–52). The study constituted two parts: a one-way pre- and post-intervention (i.e., HPV VIS education) survey (Study A) and subsequent qualitative individual interviews (Study B). For Study A, we provided the two-page-long HPV VIS for participants individually to read on a shared screen through a university virtual conference platform (i.e., Zoom). We administered online survey using Qualtrics before and after the intervention. For Study B, we performed an individual interview with the participants who had completed Study A using the same virtual conference platform. The university’s institutional review board approved this study (IRB ID#: PRO-FY2023-57).

### 2.2 Study setting

This study took place at an urban public university in the mid-south region of the U.S. The university was situated in a metropolitan area in the western region of Tennessee. The university had an enrollment of over 21,000 students at the time of the study, offering degrees from baccalaureates to doctoral levels. For characteristics of the enrolled students, approximately 60% were female, with more than half falling between the ages of 18 and 26 years old. White students accounted for about 41%, followed by African American students at 34%, Hispanic students at 8%, and Asian students at 5%. Furthermore, first-generation students constituted about 31% of the degree-seeking undergraduates. Lastly, about two thirds of the students came from in-state, while 46% were Pell grant recipients.

### 2.3 Study A

#### 2.3.1 Sampling and data collection

We performed convenience sampling to recruit study participants at the university between October 2022 and April 2023. Eligible participants should be undergraduates who were currently enrolled for the university and aged between 18 and 26 years old. For recruitment, we used advertisements of referrals, flyers, and emails. A total of 58 students participated in this study, and we sent the participants a link to an online survey that included a brief study description, a consent form, screening questions, and a baseline survey questionnaire. Among the 58 participants, 48 students were eligible and completed both the consent form and the baseline survey. We contacted these students via email to set up a Zoom-based individual virtual conference for the intervention and the post-intervention survey (as well as an individual interview) at the participants’ preferred date and time. Out of the 48 participants, 44 students were scheduled for an individual virtual conference because four students dropped out of the study due to their no more interest in the study. At the beginning of the virtual conference, we asked a participant to read the HPV VIS, which took about 5 min on average. Upon the completion of the reading, we emailed another link to the post-intervention survey for the participant to complete. Once the participant completed the post survey, we transited to an individual interview. The intervention, post-test, and interview were conducted orderly on the same day. The pre- and post-intervention surveys took approximately 15 min and 6 min, respectively, while the individual interview took about 25 min on average.

#### 2.3.2 Measures

For survey, we carefully reviewed existing literature and adopted valid measures regarding HPV vaccination for college students to collect quantitative information on knowledge, beliefs/attitudes, vaccine hesitancy, intentions, and sociodemographics (see Table 1) for comparisons in changes of the measures of interest between pre- and post-education. We excluded sociodemographic measures only from the post-intervention survey.

**Table 1:** Survey measures of interest.

Measure	# of item	Instruction/Question	Response option (re-coding)/Statement
HPV vaccine knowledge	13	“Please indicate whether the following statements are true or false”	True (= 1), Fals (= 0): Ranging from 0 to 13, with a higher score indicating greater knowledge. Only women can get infected with HPV. HPV can cause cervical cancer in women. HPV can cause cancer in areas such as the head and neck. HPV causes cancer in women only. HPV can cause genital warts. A person could have HPV for many years without knowing it. HPV is transmitted through sex. Most people infected with HPV have visible signs or symptoms of the infection. A person’s chances of getting HPV increase with the number of sexual partners they have. Nearly all sexually active people will contract HPV at some point. The HPV vaccine is only recommended for girls. Full protection against HPV requires more than 1 dose of the vaccines. The HPV vaccine is most effective if given to people who have not yet started having sex.
HPV vaccine attitudes	18	“Please indicate the extent to which you agree with each statement.”	Strongly disagree (= −2), Disagree (= −1), Agree (= +1), Strongly agree (= +2): Ranging from −36 to +36, with a higher score indicating more positive attitudes toward HPV vaccination. Having genital HPV would make it difficult for me to get a long-term sex partner. A vaccine that prevents a sexually transmitted infection is a good idea. A vaccine that prevents HPV-related cancer is a good idea. Getting the HPV vaccine would help me stay healthy. Getting the HPV vaccine would be a benefit to society. I am likely to get a genital HPV infection in my lifetime. I am likely to develop HPV-related cancer in my lifetime. I am likely to develop genital warts in my lifetime. I think the HPV vaccine might cause fertility problems. Natural immunity against HPV is better than getting the HPV vaccine. If someone gets the HPV vaccine, they may be more likely to have sex. I think the HPV vaccine is unsafe. I do not have enough information about the HPV vaccine to decide whether to get it. I think getting the HPV vaccine is beneficial. My parents would approve of my getting the HPV vaccine. My healthcare provider would approve of me getting the HPV vaccine. My religious institution would approve of my getting the HPV vaccine. The HPV vaccine is easily available.
Vaccine hesitancy	1	“Overall, how hesitant would you say you are about getting vaccinations?”	Not at all hesitant (= 1), A little hesitant (= 2), Somewhat hesitant (= 3), Very hesitant (= 4)
HPV vaccine intentions	1	“Which of the following statements best describes your intention to get vaccinated against HPV?”	I do not plan to get vaccinated ever. (= 1) I am unsure about my intention to get vaccinated. (= 2) I do not plan to get vaccinated in the next 6 months. (= 3) I plan to get vaccinated (1st shot) in the next 6 months but have not tried to schedule an appointment. (= 4) I plan to get vaccinated (1st shot) in the next month but have not tried to schedule an appointment. (= 5) I have made or tried to make an appointment to discuss HPV vaccination with my medical provider. (= 6) I have made or tried to make an appointment to get vaccinated against HPV. (= 7) I have received at least 1 shot, but do not have plans for future shots. (= 8) I have received at least 1 shot, and am scheduled to receive the next shot in the HPV vaccine series. (= 9) I have received all 3 shots of the HPV vaccine. (= 10)

##### 2.3.2.1 Vaccine knowledge

We measured HPV vaccine knowledge through adopting Harrison et al.’s 13-item true/false HPV Knowledge Questionnaire (HPV-KQ) (53).

##### 2.3.2.2 Vaccine beliefs/attitudes

To assess HPV vaccine beliefs/attitudes, we used Patel et al.’s 18-item HPV-related Beliefs/Attitudes on a four-point Likert scale (“strongly disagree,” “disagree,” “agree,” and “strongly agree”) (54).

##### 2.3.2.3 Vaccine hesitancy

We assessed the degree of vaccine hesitancy with a question (“How hesitant would you say you are about getting vaccinations?”) on a four-point Likert scale (“not at all hesitant,” “a little hesitant,” “somewhat hesitant,” or “very hesitant”).

##### 2.3.2.4 Vaccine intentions

We measured HPV vaccination intentions with one item of the HPV Vaccination Stage of Change Scale with 10 options: e.g., “I am unsure about my intention to get vaccinated,” “I do not plan to get vaccinated in the next six months,” “I plan to get vaccinated (1st shot) in the next month but have not tried to schedule an appointment,” “I have made or tried to make an appointment to get vaccinated against HPV” (54).

##### 2.3.2.5 Sociodemographic information

We measured sociodemographic characteristics, including age (year), sex (female vs. male), sexual orientation (heterosexual, bisexual, lesbian, not listed, prefer not to reply), race (Black/African American, white, Asian, Native American/American Indian), Hispanic/Latino, school year, living arrangement, marital status, employment, health insurance, HPV vaccination status (never received, unsure/do not know, and received—all recommended shots, at least 1 shot and scheduled for the next shot, or at least 1 shot but no plan), self-rate health status, and religiosity.

#### 2.3.3 Data analysis

We performed descriptive statistics to describe sociodemographic characteristics of the participants. We performed a paired t-test to assess within-group mean differences for continuous variables (i.e., knowledge and beliefs/attitudes) and Wilcoxon signed ranks test for ordinal variables (i.e., vaccine hesitancy and intentions). We conducted all statistical analyses with a 5% statistical significance level using IBM SPSS Statistics software version 29.0.1.

### 2.4 Study B

#### 2.4.1 Data collection

We developed semi-structured open-ended questions regarding HPV vaccine hesitancy, needed information for vaccine hesitancy and vaccination promotion, and preferred strategies and the potential of VR for HPV vaccination education (see Table 2). We conducted qualitative individual interviews using a Zoom-based virtual conference with 44 participants who had completed Study A. We digitally recorded all interviews via Zoom. All participants received a $40-worth electronic gift card as an honorarium after completing the interview.

**Table 2:** Interview questions.

Topic	Question
HPV vaccine hesitancy	Q: What do you think prevents college students from receiving HPV vaccines? a. PROBE: What concerns do college students have about the HPV vaccination? b. PROBE: What difficulties do college students experience in getting a HPV vaccine? c. PROBE: Are college students comfortable with talking about HPV and HPV vaccinations with friends, parents, or a healthcare provider? d. PROBE: Have you ever had a recommendation from your healthcare provider for the HPV vaccination?
Needed information	Q: Tell me about your understanding of the content in the materials (HPV VIS). a. PROBE: What information was the most interesting to you? b. PROBE: Which information was not clear to you c. PROBE: What further information do you need to answer your questions you might have? Q: Tell me about your thoughts of how the HPV VIS should be elaborated to help college students better understand HPV and the vaccination. Q: Tell me about your thoughts of which information should be developed and added to the current materials. a. PROBE: Do college students need any information that helps you receive a HPV vaccination?
Preferred strategies	Q: What do you think helps college students receive HPV vaccines? a. PROBE: What would be the most helpful for college students to participate in a HPV vaccination? c. PROBE: If so, how do you receive the information? d. PROBE: What support do college students need to receive a HPV vaccination?
The potential of virtual reality (VR)	Q: Tell me about your thoughts about using a VR platform to educate HPV and promote HPV vaccination for college students? a. PROBE: Do you think a VR simulation can be used to educate college students for HPV and HPV vaccination? b. PROBE: How could the VR-based approach be improved to influence college students’ decision on the acceptance of HPV vaccination?

#### 2.4.2 Data analysis

We analyzed the interview data transcribed verbatim using framework analysis. The framework analysis follows six phases: Familiarization with the interview data, coding, developing a working analytical framework, applying the analytical framework, charting data into the framework matrix, and interpreting the data (55). In the initial phase, three authors individually reviewed the interview data multiple times to gain familiarity. Prior to coding, the authors identified themes and sub-themes based on the interview topics. In the coding phase, the authors independently coded two interview data sets and met online to reconcile any discrepancies and finalize the codes. This coding process was then repeated for an additional three interview data sets. For coding of the reasons for HPV vaccine hesitancy, particularly, we adopted the Vaccine Hesitancy Determinants Matrix developed by WHO’s Sage Working Group that classifies factors contributing to vaccine hesitancy into individual/group influences, contextual influences, and vaccine/vaccination-specific issues (56). Subsequently, the authors developed a working analytical framework using a spreadsheet to encompass themes, sub-themes, codes, and associated quotes. Utilizing this framework, the authors independently coded the remaining interview data, cross-referencing and finalizing the matrix collectively. The interview data were then organized into the framework matrix. In the final phase, the three authors interpreted the data.

## 3 Results

### 3.1 Study A

#### 3.1.1 Sociodemographic characteristics

Table 3 presents sociodemographic characteristics of the participants. The mean age was 19 years old (SD = 1.56). Compared to the student body of the university, the participants were higher in the rates of females (71% vs. 60%), African Americans (46% vs. 34%), Hispanic/Latino (21% vs. 8%). More than two thirds were in the 1st or 2nd school years (43 and 23%). Half (50%) of the participants reported living with a parent at home. The majority (91%) were single or never married. Almost half of the participants (48%) were part-time employees. About 84% were covered by a health insurance, and 41% reported the receipt of at least shot of a HPV vaccine. Finally, two thirds (66%) reported religion as moderately to extremely important.

**Table 3:** Sociodemographic characteristics.

Variable	Total (*N* = 44)	
	*n*	%
Age	*M* = 19.4, SD = 1.56	
Sex		
Female	31	70.5
Male	13	29.5
Sexual orientation		
Heterosexual (straight)	36	81.8
Bisexual	5	11.4
Lesbian	1	2.3
Not listed	1	2.3
Prefer not to reply	1	2.3
Race		
Black or African American	20	45.5
White	15	34.1
Asian	8	18.2
Native American/American Indian	1	2.3
Hispanic or Latino		
Yes	9	20.5
No	34	77.3
Prefer not to reply	1	2.3
School year		
1st year undergraduate	19	43.2
2nd year undergraduate	10	22.7
3rd year undergraduate	9	20.5
4th year undergraduate	5	11.4
5th year undergraduate	1	2.3
Living arrangement		
Campus housing	14	31.8
With parents at home	22	50.0
Rent house of apartment	3	6.8
Own house or apartment	3	6.8
Other	2	4.5
Marital status		
Single or never married	40	90.9
Married or living as married	1	2.3
Other	3	6.8
Employment status		
Employed part time	21	47.7
Unemployed looking for work	12	27.3
Unemployed not looking for work	11	25.0
Health insurance		
Yes	37	84.1
No	7	15.9
HPV vaccination status		
Never received a vaccine	13	29.5
Unsure/Do not know	13	29.5
Received a vaccine	18	40.9
Received all recommended shots	15	34.1
Received at least 1 shot and scheduled for the next shot	2	4.5
Received at least 1 shot but no plan for a future shot	1	2.3
Self-rate health		
Excellent	9	20.5
Good	25	56.8
Fair	9	20.5
Poor	0	0.0
Very poor	1	2.3
Religiosity		
Extremely important	15	34.1
Very important	6	13.6
Moderately important	8	18.2
Slightly important	5	11.4
Not at all important	10	22.7

#### 3.1.2 Knowledge, beliefs/attitudes, vaccine hesitancy, and intentions

Table 4 shows the results from the paired t-test for the continuous variables of knowledge and beliefs/attitudes. The HPV vaccine knowledge was found to have a mean of 6.7 (*n* = 44, *SD* = 3.79) at pre-intervention, and a mean of 10.0 (*n* = 44, *SD* = 2.52) at post-intervention. The vaccine beliefs/attitudes was found to have a mean of 6.0 (*n* = 44, *SD* = 6.30) at pre-intervention, and a mean of 7.9 (*n* = 44, SD = 5.05) at post-intervention. The results indicated a significant improvement in knowledge [*t*(43) = 6.68, *p* < 0.001] and a marginal improvement in beliefs/attitudes [*t*(43) = 1.82, *p* = 0.075] regarding HPV vaccination between before and after reading the HPV VIS. Also, we performed the Wilcoxon signed ranks test for the ordinal variables of vaccine hesitancy and intentions. The vaccine hesitancy was found to have a mean rank of 6.9 at pre-intervention, and a mean rank of 6.0 at post-intervention. The vaccine intentions was found to have a mean rank of 6.8 at pre-intervention, and a mean rank of 7.3 at post-intervention. The analysis showed no significant change in vaccine hesitancy (*Z* = −0.78, *p* < 0.44) or vaccine intentions (*Z* = −0.14, *p* < 0.89) before and after the intervention.

**Table 4:** HPV vaccine knowledge and beliefs/attitudes.

	Pre-test (*N* = 44)	Post-test (*N* = 44)	*p*-value
	Mean (SD)	Mean (SD)	
HPV vaccination knowledge (min – max: 0–13)	6.7 (3.79)	10.0 (2.52)	<0.001 (one-sided)
HPV vaccine beliefs/attitudes (min – max: −36 – +36)	6.0 (6.30)	7.9 (5.05)	0.075 (two-sided)

### 3.2 Study B

#### 3.2.1 Reasons for HPV vaccine hesitancy (theme 1, research question 1)

The qualitative analysis revealed several reasons for HPV vaccine hesitancy among college students. This included individual/group influences, contextual influences, and vaccine/vaccination-specific issues (Table 5).

**Table 5:** Theme 1-reasons for HPV vaccine hesitancy.

Sub-theme	Code	Quote
Individual/group influence	A lack of awareness/knowledge of HPV and the HPV vaccination Perceived risks and no benefits of the vaccination Misperceptions of HPV infections	I’ve never heard those words—HPV, [HPV] Vaccine. (Informant File AH-8, Line 553) … even on campus I personally never had anybody talk to me about this [HPV] vaccine. (File AH-9, Line 285) I guess people do not really understand the harm that it [HPV] could cause. … So they are just not aware of the effect they can cause. (File AD-4, Line 135) I think the lack of education like I did not even know what HPV is. So this probably a lot of people out there who do not also do not know. (File AD-12, Line172) I just feel like some people think that vaccines do not usually really help too much. …I mean to prevent someone from getting it [HPV vaccines] because they just do not really think it’s that helpful. (File LJ-10, Line89) I guess some people are kind of scared of getting vaccines in general. Yeah, they might not want to get it because they are scared of it. (File AD-4, Line 145) … to be honest, I did not really like [HPV vaccination] because I was not sexually, active about that time, I wasn’t (File AH-1, Line87) You probably just like going about like thinking that you are not gonna get affected by it [HPV]. I know there is kind a like a mentality of it’s like, you know … this is not gonna happen to be like I’m like one in a 1 million, you know. (File AH-11, Line285) They think that their body can fight it [HPV] off? Naturally. (File AD-8, Line 114)
Contextual influence	Social/religious norms and stigma Parental and others’ influences Media environment A lack of transportation or no time	A religion can play into things like this [religion] as well. (File FD-3, Line 159) I want to say very hesitant [to get vaccinated against HPV]. I’m getting back to this depending on fears, or other backgrounds like religious backgrounds. Where they say oh, you cannot alter your body’s immune system, God give you this, so there might be a religious household that believe in naturalization. (File AH-4, Line 358) I do not think they are extremely comfortable doing it [talking about HPV] with just family or friends. (File FD-3, Line 168) … probably not too comfortable [talking about HPV] with their parents, because nobody really wants to talk about sex with their parents. (File LJ-4, Line 150) I realize the conversation of [HPV] vaccines became a very touchy subject for people. (File AD-11, Line 203) It’s simply because the bringing up the topic of HPV is almost like a taboo. They do not really know how to like go about it in a way. (File AD-9, Line 172) Maybe their parents aren’t willing to like [to] let them get it [a HPV vaccine]. (File AD-6, Line 748) Some, you know, families do not like the idea of being vaccinated, or just specifically like the [HPV vaccination]. (File LJ-7, Line 515) I think the availability of it I do not see it broadcasting very often, but I grew up. (File LJ-3, Line 287) I mean for me, like reading those average effects, there’s a lot on social media going around about vaccines in general (File LJ-8, Line 636) I guess, like maybe sometimes the doctors, appointments too far. And they do not have a car, or you know, like stuff like that do you think there’s any other ones. (File AH-6, Line 385) Maybe they are too busy to get the vaccine. (File FD-4, Line 487)
Vaccine Specific issues	Costs and lack of health insurance coverage Distrust in vaccines or vaccination Complex scheduling Lack of provider recommendations	I guess, like if insurance does not cover, I guess money can be an issue. (File AD-4, Line 142) …… if they [students] do not have [money], if their families are only willing to pay for that, they have to [pay] out of pocket. That’s probably a barrier [to receiving HPV vaccination]. (File AD-9, Line 157) …… and I believe they [students] are just afraid to get it [a HPV vaccine] because of the side effects of vaccinations like if they get what if they get sick, or anything like that. (File AD-13, Line 158) scheduling stuff. I know as a costume, even scheduling my own, like Doctor’s appointment takes me months, and then all has to like, push me to do it. (File AD-10, Line 142) I’m not sure if I’ve had it or not; but if I if it was recommended for me to get, I would get it. (File AD-15, Line 128) Oh, it’s recommended by a doctor, then just do it. (File AD-10, Line 158)

For the individual/group influences on HPV vaccine hesitancy, students were hesitant to receive a HPV vaccine due to a lack of awareness or knowledge of HPV and the HPV vaccination ([Informant] AH-8, [Transcript line] #553; AH-9, #285; AD-4, #135; “I’ve never heard those words—HPV, [HPV] Vaccine.”), perceived risks and no benefits of the vaccination (LJ-10, #89; AD-4, #145; “I just feel like some people think that vaccines do not usually really help too much. …I mean to prevent someone from getting it [HPV vaccines] because they just do not really think it’s that helpful.”), and misperceptions of HPV infections (AH-1, #87; AH-11, #285; AD-8, #114; “You probably just like going about like thinking that you are not gonna get affected by it [HPV]. I know there is kind a like a mentality of it’s like, you know … this is not gonna happen to be like I’m like one in a 1 million, you know.”).

For the contextual influences on the vaccine hesitancy, students stated social/religious norms and stigma (FD-3, #159; AH-4, #358; “I want to say very hesitant [to get vaccinated against HPV]. I’m getting back to this depending on fears, or other backgrounds like religious backgrounds. Where they say oh, you cannot alter your body’s immune system, God give you this, so there might be a religious household that believe in naturalization.”), parental and others’ influences (FD-3, #168; LJ-4, #150; AD-11, #203; AD-9, #172; AD-6, #748, LJ-7, #515; “Some, you know, families do not like the idea of being vaccinated, or just specifically like the [HPV vaccination]”), media environment (LJ-3, #287; LJ-8, #636; “I mean for me, like reading those average effects, there’s a lot on social media going around about vaccines in general.”), and a lack of transportation or no time (AH-6, #385; FD-4, #487; “I guess, like maybe sometimes the doctors, appointments too far. And they do not have a car, or you know, like stuff like that do you think there’s any other ones.” (File AH-6, Line 385) “Maybe they are too busy to get the vaccine.”).

Lastly, college students were hesitant for HPV vaccination due to the vaccine/vaccination-specific issues, including costs and lack of health insurance coverage (AD-4, #142; AD-9, #157; “I guess, like if insurance does not cover, I guess money can be an issue.”), distrust in vaccines or vaccination (i.e., concerns over side effects) (AD-13, #158; “…… and I believe they [students] are just afraid to get it [a HPV vaccine] because of the side effects of vaccinations like if they get what if they get sick, or anything like that.”), complex scheduling (AD-10, #142; “I know as a costume, even scheduling my own, like Doctor’s appointment takes me months, and then all has to like, push me to do it.”), and a lack of provider recommendations (AD-15, #128; AD-10, #158; “I’m not sure if I’ve had it or not; but if I if it was recommended for me to get, I would get it.”).

#### 3.2.2 Needed information for HPV vaccination (theme 2, research question 2)

The analysis also revealed college students’ needed information for reducing HPV vaccine hesitancy and facilitating the uptake (Table 6). This information included places for HPV vaccination, concrete descriptions of the virus and the vaccination, and other practical information.

**Table 6:** Theme 2-needed information for HPV vaccination.

Sub-theme	Code	Quote
Information needed for HPV vaccine hesitancy and the vaccination	Places to get vaccinated against HPV Concrete descriptions of HPV and the vaccination Other practical information	I do want to know like. How would you go about trying to get the HPV vaccine? (File AD-9, Line 146) I think that including where, to get the vaccine would help, because I do not know if they offered at Walgreens or any other of those type of health companies do… (File LJ-3, Line 454) Also probably like how it does cause cancer and stuff. (File AH-2, Line 648) Where that like originates from and like what it like really is. I guess that makes sense and also probably like how it does cause cancer and stuff. (File LJ-9, Line100) How the vaccine works. I think that’s where a lot of like, Miss. Yeah, I think that’s where a lot of like people assuming things comes from, and like with any vaccine like, especially with the Covid vaccine. It was just like n0 0ne knew how it came to be, or like how it works. And so I feel like by giving a more thorough explanation of that we’ll allow like (File LJ-8, Line1027) I think I think so statistics are good. I think it’s you know. I can definitely play to the advanced, because you can definitely list, like how many people have gone like cervical, cancer, or any type of cancer. As well. You can say like how many people like live with HPV without even knowing it, like, I think that in itself, hey, people see like these like if they are able to like have like a number put in there to it’s easier for them to compromise in their to Comprehend it in their mind. So it’s I actually think it’s a really good idea. You know. (File AH-1, Line704) I guess the material It would be nice if it showed like the average cost of the vaccine or the average cost of the vaccine for people with or without insurance. (File FD-3 line 258) What then they can do is include like expected price. They like, we are co-paying. If you have to pay it out of pocket. (File FD-9, Line 637)
Suggestions for improvements in existing information	Visualization Readability	Probably not cause it was like, yeah, Maybe if it was like colorful, or more like graphic design, like just not so like Black, and white, just straight, text. (File AH-6, Line714) This point maybe color or illustration. and then a percentage like with pamphlets. You know how there is a percentage like. (File AD-11 Line248) I think statistics would probably help especially if they are like big like it said almost everyone would contract. And infection in their life, and like something like that would probably be like good to emphasize. (File AH-6, Line 670) I think that it should just be something simplified where we, as I mean people that is not in the medical field, can read and understand. (File FD-10, Line 516)

More specifically, students stated that they sought to receive information on where to get vaccinated (AD-9, #146; LJ-3, #454; “I think that including where, to get the vaccine would help, because I do not know if they offered at Walgreens or any other of those type of health companies do……”). Students also mentioned that the information should include the process of HPV infection and its consequences, a mechanism in which a vaccine prevents against HPV and relevant cancers, and costs related to the vaccination (AH-2, #648; LJ-8, #1027; AH-1, #704; FD-3 #258; FD-9, #637; “How the vaccine works. I think that’s where a lot of like, Miss. Yeah, I think that’s where a lot of like people assuming things comes from, and like with any vaccine like, especially with the Covid vaccine.”). Additionally, students offered suggestions for improving the existing HPV VIS. Students indicated that the HPV VIS should include more visuals (e.g., statistics, graphics, illustrations), and its content should be simple to enhance readability (e.g., plain language avoiding medical jargons) (AH-6, #714; AD-11, #248; FD-10, #516; AH-6, #670; “Probably not cause it was like, yeah, Maybe if it was like colorful, or more like graphic design, like just not so like Black, and white, just straight, text.”).

#### 3.2.3 Preferred strategies and the potential of VR for the vaccination promotion (theme 3, research question 3)

Students suggested several strategies for facilitating HPV vaccination among college students (Table 7).

**Table 7:** Preferred strategies and the potential of VR for HPV vaccination promotion.

Sub-theme	Code	Quote
Strategies for HPV vaccination promotion	Communication with friends or parents Provider recommendations Financial/social support	I feel like they are so influenced by peers. (File AD-11, Line 236) I would mostly say, social norms of what they [Students] hang around. (File AH-5, Line 300) The people I hang out with. They [Students] ‘re pretty comfortable talking about stuff like that [HPV]. (File LJ-6, Line 547) Because my doctors are always like telling me about random stuff, and I just agree with whatever they say. (File AD-6, Line 658) I have full trust in my health care team. I have a lot of medical history. (File LJ-7, Line 665) I’ll just have to talk with my doctor about it [HPV Vaccine], because I do not know what condition a human has to be in for it [HPV Vaccine] to be safe or not. (File FD-3, Line 182) Yeah, probably financial support…. I do not know how much it [HPV Vaccine] costs to go to the office like I do not cause I’m still under my mom’s insurance. (File AH-10, Line 308) As for financial support, maybe like covering costs over like vaccines, If that’s a problem for some students. (File LJ-7, Line 783) They’ll also need financial support, because I’m sure people without insurance. (File FD-3, Line 203) Family support is one thing how would your parents feel about you, or how would your parents agree. (File FD-3, Line 223) So maybe they could have a friend with them [students], a parent they could like, kind of help them through that[fear about vaccine]. (File LJ-4, Line 177)
Channels of delivery of HPV vaccination information	Email Website Social media Campaigns on campus In-class session	Like regular emails about it [HPV Vaccine], like, how they did with Covid, I feel like that, would help a lot. (File AH-1, Line 516) Any websites like I said the CDC. They should have it [information] to where like maybe in better terms. (File AD-8, Line 146) I think the University Health Center has an Instagram or something around that if they were to make a post about HPV. (File AH-2, Line 538) Let us say someone just has a booth every month like outside the [University] just talking about this[HPV Vaccination]. It’s just like, Hey, you should get this. (File AH-8, Line 643) Stands out in college campus if they had like a stand that said like Hpv vaccine and they had information telling people to come over. (File AD-12, Line 190) Maybe focusing on more like smaller communities within the University of like we were having like an info session. (File LJ-8, Line 903) It should absolutely just like me talked about just like a singular session, like a a giant session. (File AD-9, Line 180)
VR-based education on HPV vaccination	Positive attitudes Game-based learning Scenario-based Simulations	I think it’s Pv. in anything in technology. So if you put on anything I want to be more efficient one I would understand. (File AD-5, Line 229) Absolutely. I think it’s a brilliant idea, because VR is like the future. So if you want to try to get more people like exposed to this information, doing a way where like you would like the future. And technology is advancing, and a lot of people will be interested in VR and be like, oh, what does that look like? Or just one experiences, so that you do, that while you also expose them to this information, also like they’ll Skyrocket. (File AH-4, Line 778) I would think some sort of like trivia, I guess, like a trivia game. I’m: not really sure. I’m: not too like technical, but some sort of game to get people to understand the knowledge and concepts. (File LJ-6, Line801) Yeah, I think, like I was saying before, with the whole consequences thing if you see it up front, and you see it happening, then, like you’ll be more tempted to take it. (File Ad-10, Line 201) If there is a way to like showcase Somebody’s life like not getting the vaccine, but then get any HPV. And like, I guess, like the most the worst-case scenario from it. (File AD-9, Line 235) like sort of if there was like a model or a big like. The different little this is gonna sound sort of like the little particles and stuff of the body that, like you know all that. And it just if it was a simulation, or whatever showing how you know the HPV virus like comes in like attacks, the cells, or something like that. And then it sort of shows how the entire person like I do not know, starts coughing, or whatever the symptoms may be if that was all sort of. (File AD-10, Line 191)

These strategies included communications with a friend or parent (AD-11, #236; AH-5, #300; LJ-6, #547; “I would mostly say, social norms of what they [Students] hang around.”), provider recommendations (AD-6, #658; LJ-7, #665; FD-3, #182; “I’ll just have to talk with my doctor about it [HPV Vaccine], because I do not know what condition a human has to be in for it [HPV Vaccine] to be safe or not.”), and financial or social support (AH-10, #308; LJ-7, #783; FD-3, #203; FD-3, #223; LJ-4, #177; “They’ll also need financial support, because I’m sure people without insurance.”). Students also stated that their preferred channels of delivery of HPV vaccination information included email, website, or social media (AH-1, #516; AD-8, #146; AH-2, #538; “Like regular emails about it [HPV Vaccine], like, how they did with Covid, I feel like that, would help a lot.”), campus-wide campaign, and in-class session (AH-8, #643; AD-12, #190; LJ-8, #903; AD-9, #180; “Let us say someone just has a booth every month like outside the [University] just talking about this [HPV Vaccination]. It’s just like, Hey, you should get this.”).

Lastly, students mentioned their thoughts and opinions of using VR as an education platform for reducing HPV vaccination hesitancy and increasing the uptake (Table 7). Most of the students revealed the positive attitudes toward the use of VR, stating that VR would be an attractive (“*cool*”), effective platform for HPV vaccination education among college students (AD-5, #229; AH-4, #778; “I think it’s a brilliant idea, because VR is like the future. So if you want to try to get more people like exposed to this information, doing a way where like you would like the future. And technology is advancing, and a lot of people will be interested in VR and be like, oh, what does that look like? Or just one experiences, so that you do, that while you also expose them to this information, also like they’ll Skyrocket.”). To maximize the education outcomes, students added that the VR-based vaccination education should involve game-based learning (LJ-6, #801) and scenario-based simulations guiding a vaccination procedure (AD-10, #201; AD-9, #235; AD-10, #191; “I think, like I was saying before, with the whole consequences thing if you see it up front, and you see it happening, then, like you’ll be more tempted to take it.”).

## 4 Discussion

Using a mixed-methods approach, we examined the effects of a conventional HPV vaccination education approach using the HPV VIS in advancing HPV vaccination outcomes including knowledge, beliefs/attitudes, vaccine hesitancy, and intentions among college students. We also assessed college students’ perceived reasons for HPV vaccine hesitancy, needed information, and preferred strategies including VR technology for HPV vaccination education. The findings provided essential information on designing HPV vaccination information or messaging focused on college students’ vaccine hesitancy. Also, the findings offered several implications for future research on interventional strategies for increasing HPV vaccine acceptance among college students.

Firstly, we found that the exposure to the HPV VIS among college students was effective in improving their knowledge regarding HPV vaccination, but no effect was observed in beliefs/attitudes, vaccine hesitancy, and intentions. This finding supports the fundamental role that conventional vaccination education including the VIS plays in increasing knowledge of HPV vaccination among college students (36). As consistent with the existing literature, however, this finding also implies that the conventional educational approach may have a limited influence on other HPV vaccination outcomes (i.e., beliefs/attitudes, vaccine hesitancy, and intentions) in this group which mediate the link between knowledge and uptake (23). According to theoretical frameworks of health behavior change (including health belief model and theory of planned behavior), an intention to engage in vaccination is determined by a person’s beliefs, attitudes, and subjective norms at a specific context and time (43, 57). Accordingly, it is critical for HPV vaccination education for college students to employ multi-interventional components that address their vaccine-related beliefs and attitudes associated particularly with their social norms. Future research should examine an optimized, theory-informed combination of these interventional components. These findings can help public health researchers and practitioners better understand the paths from the beliefs or attitudes to intentions, resulting eventually in the uptake among college students. In the paths, it is also important to first investigate college students’ information needs and specific reasons for the vaccine hesitancy and then design content and messaging tailored to effectively mitigate their vaccine hesitancy (58).

Secondly, we identified primary reasons for HPV vaccine hesitancy that college students perceived. While these reasons mostly are consistent with the existing literature, particularly, our findings underscore the context specificity of HPV vaccine hesitancy (i.e., social/religious norms and stigma and parental and others’ influences) in this group. Many students in our study mentioned that they felt embarrassment or difficulties of openly discussing HPV-related topics with parents and others in milieu of the mid-south region of the U.S. Particularly, this region has been widely affected by religious and political norms in which HPV is perceived as a taboo. In this study nearly two thirds reported that religion is moderately to extremely important. Vaccine hesitancy related to social/religious norms might contribute to consistently reported lower rates of HPV vaccination, compared to different U.S. regions (59), along with high rates of HPV prevalence, HPV-associated cancers, and cervical cancer mortality (60) in this region. While researchers and practitioners need to consider the identified reasons for HPV vaccine hesitancy in developing interventions, the interventional efforts should address the context-specific reasons for HPV vaccine hesitancy in college students.

Next, we identified college students’ needed information and preferred strategies for HPV vaccination promotion including channels of vaccination information delivery. The findings highlight the urgent need of developing specific and practical information for college students’ HPV vaccination promotion such as places for vaccination and vaccination procedure. In particular, as a HPV vaccine was not available to students on campus at the time of this research, interventional efforts should involve stakeholders on HPV vaccination at the university including a health student center in the initial stage of research. The findings also imply the importance of influences by peers, parents, and healthcare providers on vaccination-related decision making. This suggests that interventional components should leverage these influences when they design content and messaging related to the vaccination for college students. These efforts will enhance the vaccine accessibility in terms of locations, costs, and health insurance as well as the acceptability of the targeted or tailored information on HPV vaccination.

Finally, we found that college students viewed VR as a valuable technology for HPV vaccination education. Prior studies also showed that VR-based vaccination education had a positive impact on improving vaccination attitudes and intentions among the adult populations. For example, Vandeweerdt et al. found that VR was more effective in increasing COVID-19 vaccination intentions among adults, compared to texts and images (61). Nowak et al. also used a VIS paired with a VR-based simulation improved influenza vaccine-related attitudes and intentions among vaccine-avoidant adults aged 18 to 49 years (62). Furthermore, a recent study found that VR simulations significantly increased perceived efficacy to influenza vaccination and vaccination intention for both protecting the self and others in the community among college students in Taiwan (63). In these studies, VR played a significant role in making participants’ vaccination experience more immediate, engaging, and informative within mediated immersive environments than did the conventional approaches (61, 62, 64). A systematic review indicated that VR’s unique features, such as embodiment, realism, and interactivity, enable users to overcome their psychological barriers to a target behavior, leading to an increased likelihood of behavior change (48). Similarly, because vaccine hesitancy poses a wide array of psychological barriers (e.g., social/religious norms and stigma) to HPV vaccination, these findings imply the potential of VR to mitigate hesitancy for HPV vaccination, serving as an effective educational platform for improving HPV vaccination outcomes. Next studies should examine the acceptability and feasibility for the use of VR in the context of HPV vaccination among college students. As the distinct gap in HPV vaccine coverage by gender has persisted, future research should also examine VR-based educational interventions for addressing gender-specific hesitancy for HPV vaccination.

### 4.1 Limitations

We conducted a mixed-methods study to obtain comprehensive data, but the findings of this study might include some limitations. As the study employed non-probability sampling and a one-way study design, the findings may not be generalizable to other populations or settings. Additionally, the data was based on self-report which could introduce some recall bias. While individual interviews with a sample size of 44 achieved saturation to enhance the trustworthiness of the findings, the pre- and post-intervention survey with this sample size might not fully capture the effect of the HPV VIS in the changes in the beliefs/attitudes, vaccine hesitancy and intentions. Future studies using probability sampling along with a comparison group can offer a better understanding of the role of conventional vaccination education in college students (e.g., unvaccinated vs. vaccinated participants). Finally, this study explored HPV vaccine hesitancy in college students employing a framework analysis approach. Different qualitative analytic approaches (e.g., content analysis, thematic analysis) may reveal different themes and codes.

## 5 Conclusion

This mixed methods research informed effective educational approaches to HPV vaccination promotion targeted toward college students. The pre- and post-intervention survey found positive effects of a conventional vaccination education using the HPV VIS in increasing knowledge for college students. However conventional vaccination education also showed limitations in influencing beliefs/attitudes, vaccine hesitancy and intentions in this group. Also, the qualitative individual interviews identified primary reasons for HPV vaccine hesitancy among college students and needed information, for HPV vaccination education which can guide future interventions. These findings offer useful implications for generating strategies for educating and facilitating college students to increase HPV vaccination acceptance particularly through addressing their reasons for vaccine hesitancy. Also, the findings provide essential insight for future interventions using the potential of a VR-based HPV education for improving the vaccination outcomes in college students.

## Data availability statement

The original contributions presented in the study are included in the article/supplementary material, further inquiries can be directed to the corresponding authors.

## Ethics statement

The studies involving humans were approved by the Institutional Review Board of the University of Memphis. The studies were conducted in accordance with the local legislation and institutional requirements. The participants provided their written informed consent to participate in this study.

## Author contributions

SY: Formal analysis, Writing – original draft. HK: Formal analysis, Writing – original draft. JA: Formal analysis, Writing – original draft. SJ: Conceptualization, Data curation, Formal analysis, Funding acquisition, Investigation, Methodology, Project administration, Resources, Software, Supervision, Validation, Visualization, Writing – original draft, Writing – review & editing.
